# Treatment of Electroplating Wastewater Using NF pH-Stable Membranes: Characterization and Application

**DOI:** 10.3390/membranes10120399

**Published:** 2020-12-06

**Authors:** Ignacio Hegoburu, Karina Listiarini Zedda, Svetlozar Velizarov

**Affiliations:** 1 Associated Laboratory for Green Chemistry—Clean Technologies and Processes (LAQV), REQUIMTE, Chemistry Department, FCT, Universidade Nova de Lisboa, 2829-516 Caparica, Portugal; ignaciohegoburu@gmail.com; 2 LANXESS—IAB Ionenaustauscher GmbH Bitterfeld, 06803 Bitterfeld-Wolfen, Germany

**Keywords:** nanofiltration, electroplating, extreme pH, acid recovery, Donnan effect

## Abstract

Industrial adoption of nanofiltration (NF) for treatment of low-pH wastewater is hindered by the limited membrane lifetime at strongly acidic conditions. In this study, the electroplating wastewater (EPWW) filtration performance of a novel pH-stable NF membrane is compared against a commercial NF membrane and a reverse osmosis (RO) membrane. The presented membrane is relatively hydrophobic and has its isoelectric point (IEP) at pH 4.1, with a high and positive zeta potential of +10 mV at pH 3. A novel method was developed to determine the molecular weight cut-off (MWCO) at a pH of 2, with a finding that the membrane maintains the same MWCO (~500 Da) as under neutral pH operating conditions, whereas the commercial membrane significantly increases it. In crossflow filtration experiments with simulated EPWW, rejections above 75% are observed for all heavy metals (compared to only 30% of the commercial membrane), while keeping the same pH in the feed and permeate. Despite the relatively lower permeance of the prepared membrane (~1 L/(m^2^·h·bar) versus ~4 L/(m^2^·h·bar) of the commercial membrane), its high heavy metals rejection coupled with a very low acid rejection makes it suitable for acid recovery applications.

## 1. Introduction

Membranes have proven to be a key technology to achieve process intensification in unit operations concerning separation and purification. In recent decades, nanofiltration (NF) has emerged as an alternative to reverse osmosis (RO) and ultrafiltration (UF) membranes, providing higher selectivity than UF with a lower pressure gradient than RO. The key distinguishing aspect of NF is a potent separation of inorganic salts and small organic molecules, via low rejection of monovalent ions (20–70%), high rejection of multivalent species (>80%) and uncharged solutes with molecular weights (MWs) from 100 to 1000 Da, together with a higher flux than their RO counterparts [1].

These characteristics have enabled the adoption of NF by a multitude of applications, including water and wastewater treatment, pharmaceutical and biotechnology, and food engineering. One of the main reasons behind the success of polymeric membranes in so diverse applications is their optimization to deal with harsh process conditions, such as high temperature, organic solvents, extreme pH and an oxidative environment. Such development can be seen in the history of polymeric membranes: the first commercial cellulose acetate (CA) membranes in the 1970s were stable only within a pH range of 4–8, whereas current polyamide composite membranes can be operated continuously at pH values between 2 and 12 [2].

However, that pH window is still too narrow for applications which involve highly acidic or highly alkaline solutions. One explanation for the lack of pH stability of conventional polyamide thin film composite (TFC) membranes is associated with the nature of the amide bonds. These bonds can be hydrolyzed in the presence of an acid or base catalyst, which turns the carbonyl group more susceptible to a nucleophilic attack [3]. Therefore, current research into pH-stable polymeric NF membranes is focused on the development of materials which can resist the extreme pH condition during longer operation times without compromising the selectivity and permeability performance.

Polymers which are relatively stable towards acids and that can be engineered into membranes include polyolefins such as polyethylene and polypropylene, polysulfone (PS), polyethersulfone (PES) and polyether ketones. Unfortunately, obtaining the right balance between solute rejection and water and acid permeability (in order to yield a purified acid stream in the permeate side) is not straightforward [4]. For example, by the 1980s, it was already known that PS and PES could be casted into asymmetric membranes with stability to operate at pH from 0 to 14 even at relatively high temperatures of up to 80 °C. However, their molecular weight cut-offs (MWCOs) (between 1000 and 2000 Da) were still too high to enable selective separation of low-molecular weight compounds (e.g., monosaccharides and disaccharides). Besides, these membranes easily fouled during industrial application, with a consequent loss of permeate flux. One approach to minimize fouling consisted in casting the membrane with a compatible hydrophilic polymer [5].

The previous difficulties explain the currently limited offer of commercial pH-stable NF membranes [3]. Table 1 presents a summary of such membranes, together with their main characteristics. It shows that most commercial pH-stable membranes are polymeric. Despite their superior chemical and thermal stability [6], ceramic NF membranes are much less commercialized. This is mainly due to their comparatively high cost, which results in a complex manufacturing process and lower packing densities of ceramic modules, thus requiring a larger footprint and higher capital expenditure. Table 2 presents a non-exhaustive list of recently developed pH-stable polymeric NF membranes. In the preparation of all membranes in this list, synthetic routes involving the formation of polyamide bonds were avoided in order to prevent their pH-promoted degradation. Different materials have been applied, ranging from polyamines to functionalized ketones and poly(vinylidene fluoride) (PVDF). However, one major limitation persists among these membranes, as none of them can achieve an MWCO below 500 Da. Tighter NF membranes are required to separate low-molecular weight compounds. 

There are many industrial applications which could benefit from using of pH-stable NF membranes. Examples include treatment of streams such as acid mine drainage [21,22,23,24], rare-earth elements recovery from mining residues [25], purification of rinsing water of electroplating industries and effluents from anodizing, galvanizing, brightening and pickling processes [26,27,28]. In all these cases, the strong acidic conditions keep the metals present in solution.

A previous in-house study devoted to the effect of pH and membrane charge on the nanofiltration of acid mine drainage showed promising results in terms of simultaneous high heavy metals rejection and low rejection of sulfates, which can be utilized as a way of separating the metals from the accompanying sulfur-containing compounds [29]. The transport of inorganic species coupled to changes in their speciation has been studied by López et al. [30], who also report a strong decrease in the rejection of total sulphate (SO_4_^2−^/HSO_4_^−^) as the fraction of single-charged hydrogen sulphate (HSO_4_^−^) in the feed increases.

A less studied source of acidic wastewater which is also rich in heavy metals and sulfates is the electroplating industry. Electroplating refers to the electrochemical coating process in which metals are reduced and thus deposited on top of a substrate in order to provide the material with specific properties. Electroplating wastewater (EPWW) is generated as a result of water rinsing of the coated pieces in between each process step and at the end of the cycle to remove all traces of chemicals that remain on the surface [31]. Heavy metals such as chromium, copper, zinc, nickel and iron typically present in EPWW pose a threat to both human health and the environment even at very low concentrations, as a result of their high toxicity and potential for bioaccumulation [32]. A more detailed discussion of the typical EPWW composition is presented in Section 2.6.1.

A conventional method to treat EPWW is precipitation of heavy metals as hydroxides. Such an approach requires pH adjustment (with the consequent consumption of lime), is not fully selective and generates large amounts of solid sludge for disposal [33]. Therefore, big room for improvement exists. Compared to other alternatives such as electrolytic deposition, electrodialysis and RO, NF offers a relatively lower energy consumption [30]. This is due to the fact that NF membranes show low rejection of hydronium ions, leading to their similar concentrations on the permeate and retentate sides. There is then a lower contribution of hydronium ions to the differential osmotic pressure, resulting in lower pumping costs than those for RO.

Therefore, acid-stable NF membranes are great candidates to help close the loop of EPWW treatment since they can reject the multivalent cations, thus concentrating them on the retentate and simplifying their further recovery by downstream processes. On the other hand, hydronium ions can selectively permeate through the membrane together with anions, yielding a purified acidic stream in the permeate which can be reused in the process.

The present work is dedicated to investigating the performance of a novel pH-stable NF polymeric membrane for its potential application in treating electroplating wastewater. The membrane was characterized in terms of its surface charge, morphology, hydrophilicity, MWCO and EPWW filtration performance. A benchmark study against a commercial pH-stable NF polymeric membrane was performed in relation to their performance in treating EPWW. An RO membrane was used in some experiments for comparison purposes.

Most previous studies on pH-stable NF have tested the filtration performance at neutral pH after immersion of membrane coupons in acidic or alkaline solutions, assuming that changes occurring in the membrane are irreversible [3,10,12,19]. However, it has been reported that the membrane structure can change reversibly with a changing pH [34], which implies that tests at neutral pH may not be representative of the actual membrane performance during operation at extreme pH conditions. For this reason, in this work, crossflow filtration experiments were performed at the actual pH value of the targeted application (treatment of EPWW), including not only salt rejection experiments but also MWCO determination. This last point is particularly challenging, since samples at extreme pH cannot be readily analyzed by standard gel permeation chromatography (GPC). Therefore, a novel method based on organic extraction of polyethylene glycol (PEG) markers (used for MWCO determination) from aqueous samples is hereby presented, which is an alternative to a previous methodology proposed by Dalwani et al. [34].

## 2. Materials and Methods

### 2.1. Membranes

Membrane filtration experiments were carried out using two pH-stable NF membranes (Membrane A and Membrane B), and, for the purpose of comparison, an RO membrane (Membrane C) was also included in the filtration of single salts. Membrane A is a novel in-house manufactured asymmetric membrane, which consists of a hydrophilized PS selective layer obtained via phase inversion and thermally crosslinked, supported on non-woven fabric. The nature of the hydrophilizing agent is under know-how protection. Membrane B is made of sulfonated PES and is commercialized as a pH-stable membrane able to withstand continuous operation within the pH range 0–14. Membrane C is a TFC RO membrane manufactured by Lanxess. It has a polyamide skin layer supported on a polysulfone substrate and can operate continuously at pH 2–11, with it being possible to expose it during short periods of time to more extreme pH conditions (pH 1 or pH 12) for cleaning purposes.

### 2.2. Chemicals and Solutions

All solutes were used as purchased without further purification. A weighing balance (Mettler Toledo XS6002S) was used, and solutes were added directly to deionized (DI) water (approximately 1.0 µS/cm and pH 5.8) to make up the solution. The solution of chromium (III) sulfate needed heating and stirring at 80 °C for several hours until it reached complete dissolution. Polyethylene glycol (PEG) marker molecules of higher molecular weight (600, 1000, 2000, 3000 MW) required stirring for some minutes to be fully dissolved. The chemicals used in this study are summarized in Table 3.

### 2.3. Filtration Tests

**Figure 1:**
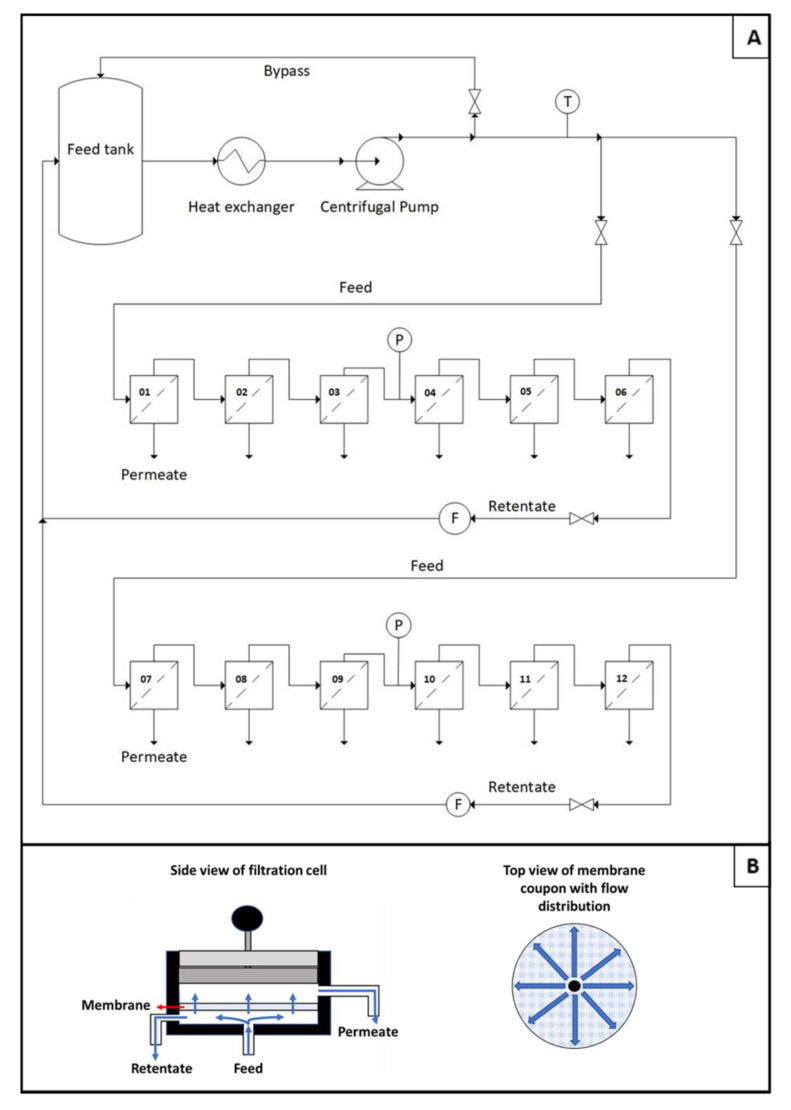
Crossflow filtration unit used to perform the permeation tests. (**A**) Flow sheet of equipment. (**B**) Left: scheme of filtration cell design; right: feed flow distribution on the membrane coupon.

All filtration tests were performed using an in-house custom-built flat sheet crossflow filtration membrane coupon tester (CT), as the one depicted in Figure 1. The equipment consists of 12 filtration cells, arranged in two benches of 6 cells each. The cells fit circular membrane coupons of 5.8 cm diameter (area of 26.55 cm^2^) and the permeate can be individually collected in each cell. The filtration cells are arranged in a series configuration (feed originates from the retentate of the upstream adjacent cell), but due to the very small recovery rate, a change in feed concentration between the first and last cells is deemed negligible.

Filtration experiments were carried out in batch recirculation mode, i.e., the retentate was recirculated and mixed with the feed tank. When the permeate was not being collected, it was also recirculated. A plastic tank of 60 L was used to store the feed, a high-pressure centrifugal pump was used to provide the driving force and a heat exchanger was used to keep the feed temperature controlled. Pressure was measured using a pressure gauge at the retentate of the third filtration cell of each bench. This pressure was taken as the imposed operating pressure, ∆P, assuming that the permeate side pressure is negligible. Flow rate was measured by the flow meter after the last filtration cell and three valves were used to adjust its value to the operating conditions.

Unless otherwise stated, all permeation tests were conducted at a feed flow rate of 4 L/min under an applied pressure of 10 bar at the feed side and at a controlled temperature of 25 °C. The tangential crossflow velocity can be calculated as the ratio of the feed flow rate and the cross-sectional area for water flow. Due to the cell geometry, the crossflow velocity tangential to the membrane surface is higher in the center of the coupon and decreases as the flow lines approach the external diameter of the membrane. At this outer point, the minimal crossflow tangential velocity is 0.18 m/s. At these conditions, a pressure drop of 0.25 +/− 0.05 bar is obtained in each cell. Since the 10-bar feed pressure is measured between the third and fourth filtration cells, this means that the first cell has a feed pressure of 10.75 bar, whereas the sixth cell has a lower feed pressure of 9.25 bar.

For each set of experiments, fresh membrane coupons were used, and before and after each experimental set, the coupons were tested with a feed solution containing 2000 ppm of MgSO_4_ at pH 7. The main advantage of the CT installation utilized is that it enables the simultaneous testing of up to 12 membrane samples. In each experimental set, at least 3 samples of each membrane type were tested, enabling the calculation of the results’ standard deviation. Whenever pH adjustment was needed, 10 wt.% aqueous solutions of NaOH or H_2_SO_4_were added, as will be specified for each experimental set.

Before taking the first permeate collection, the system was left running at full recirculation mode for at least 90 min to achieve membrane compaction. For each experimental run, three successive permeate collections were conducted, with 60 min stabilization time in between each collection.

The increase in salts concentration in the feed throughout the experiment is small, as the volume loss by permeate sampling (approximately 12 × 50 mL) represents only 1% of the feed volume. However, to minimize the source of error, the feed was sampled before and after each permeate collection and these two samples were mixed. The mixed sample was the one used for the determination of the feed composition.

### 2.4. Membrane Performance Calculations

In all filtration experiments, rejection was calculated using Equation (1).

(1) $$R_{i,\;observed}=\left(1-\frac{{cP}_{i}}{{cf}_{i}}\right)$$

By efficient cell design and appropriate hydrodynamic conditions (discussed in a previous work [29]), concentration polarization effects are deemed negligible. Therefore, the calculated observed rejection is expected to be very similar to the real rejection value.

Permeate flux was calculated through Equation (2) as

(2) $$J_{v}=\frac{V}{A_{m}\cdot t},$$

where the volume of the permeate sample was obtained from the weighed mass of the permeate, taking the density of water as 997 kg/m^3^ (a reasonable assumption for dilute solutions like the ones in this work). The osmotic pressure of the feed and permeate was calculated from the van’t Hoff equation (Equation (3)).

(3) $$ᴨ=\overset{n}{\underset{i=1}{\sum }}C_{i}\cdot R\cdot T$$

The molar concentration of each solute was determined using the corresponding analytical method, and the contribution of hydronium ions to the osmotic pressure was considered in all the low-pH experiments (pH < 4).

### 2.5. MWCO Determination

MWCO is usually determined using probe molecules of different molecular weights and is defined as the value for which 90% rejection of the probe molecule is achieved. When neutral marker molecules are used, MWCO can be used to analyze to which extent steric exclusion contributes to the overall rejection performance of the membrane.

PEGs fractions are used, since they are neutral molecules with high solubility in water and with a large spectrum of molecular weights covering the whole interval of potential MWCO values under study. Determination of their concentration in the feed and permeate can be achieved through GPC, in which the different retention times within the column for each molecular weight probe result in the separation of the peaks in an elugram.

Most scientific literature deals with MWCO determination at neutral pH and not in the pH extremes which are relevant for pH-stable membranes. Dalwani et al. developed a method to determine the MWCO in filtration tests carried out at low and high pH, using PEG as marker molecules after having verified its stability at pH 1 and 13. Basically, the authors propose neutralizing the feed and permeate samples collected during the filtration test of the marker molecules. However, this neutralization step yields a quite high salt concentration in the samples, which results in a salt peak in the GPC chromatograms that overlaps with the PEG peaks. The authors propose adding this salt to the GPC eluent in the same concentration as in the sample. In this way, they modify the baseline of the GPC determination and the salt peak is no longer detected [34].

In order to avoid the need of adapting the GPC eluent, an alternative method is hereby presented. In this approach, acidic feed and permeate samples are neutralized with 0.1 M NaOH. Then, the ionic strength of all samples is equalized by adding Na_2_SO_4_, and three consecutive extractions with chloroform are performed (in each extraction, 50 mL of chloroform is used per 20 mL of aqueous sample), in order to transfer the PEG molecules in the aqueous sample to the organic phase. The three extracted fractions are then mixed, and the organic solvent is evaporated using a rotary evaporator at 100 mbar and 40 °C. The remaining viscous residue is then dissolved in the GPC eluent, to finally obtain the corresponding elugram used as input in the sieve curve analysis.

Before application, the method was validated by preparing a mixture of PEGs of different MWs at neutral pH. This mixture was processed in two ways: (i) through the organic extraction process previously described; and (ii) by simply dissolving the mixture in the GPC eluent. Then, chromatograms were obtained for the mixture treated in each of the two ways. These chromatograms are completely coincident, thus proving that the organic extraction does not affect the molecular weight distribution of PEG.

**Figure 2:**
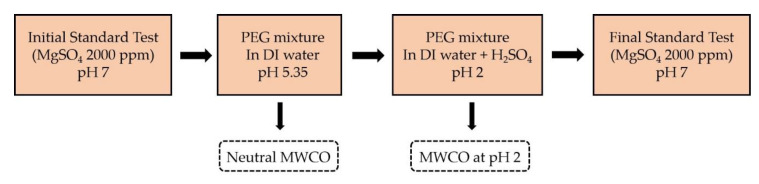
Scheme of crossflow filtration experiments performed with the coupon tester (CT) at standard conditions.

The implementation of the MWCO determination experiments is presented in Figure 2. It shows the series of filtration tests performed to obtain the MWCO at pH 2. The first step consisted in a standard test with MgSO_4_ 2000 ppm, using 6 coupons of Membrane A and 6 coupons of Membrane B in the CT equipment. Then, the CT was cleaned, and a new feed was prepared in the 60 L tank, consisting of DI water and a mixture of PEGs (200/300/400/600/1000/2000/3000 Da), with a concentration of 1 g/L of each PEG size. The spontaneous pH arising from the dissolution of the PEGs in DI water was 5.35 ± 0.05, as no NaOH was added to avoid the presence of ions that could potentially interfere with the rejection behavior of PEGs. The crossflow filtration test of this solution was performed at standard conditions, and samples of feed and permeates were collected to assess the MWCO.

Then, H_2_SO_4_was added to the CT feed tank to shift the pH of the PEG mixture to 2. The crossflow filtration test was repeated and feed and permeate samples were collected to evaluate the MWCO at a pH of 2. Finally, the CT was cleaned and a new standard test with MgSO_4_ 2000 ppm was performed to compare the membrane performance with the original values before filtration of the PEG molecules.

### 2.6. Model EPWW

#### 2.6.1. Criteria for Defining Solution Composition

EPWW was chosen as a case study to evaluate the feasibility of applying pH-stable NF membranes for the recovery of heavy metals and purified acid. To better control the experimental conditions, a model EPWW rather than an actual effluent was used. To design the model EPWW, different literature references were consulted, so as to keep the conditions of the simulated wastewater relevant and representative of the actual process.

The composition of EPWW can vary widely depending on the specific surface treatment and the rinsing method [35]. However, it typically contains low organic matter and heavy metals concentrations in the order of several ppm. The work from Wei et al. presents the composition of a sample of real EPWW obtained from an electroplating plant in China [27]. These values are compared to those reported by Wang et al. [26] in Table 4, who also used EPWW from a Chinese plant.

Both samples have a similar pH, but the one used by Wei et al. contains a significantly higher concentration of all ions. Clearly, this sample will be more challenging to treat by means of NF in terms of meeting the maximum admissible levels of heavy metals in the permeate. For this reason, the target concentrations pursued in the simulated EPWW are close to those presented in Wei’s work.

However, some simplifications are introduced to the composition of the simulated EPWW. For example, no potassium cations are added, so that the only monovalent cation is sodium. Similarly, among the anions, the presence of nitrates and nitrites is neglected, so that chlorides are the only monovalent anions. The reason behind these modifications is to better understand the rejection of heavy metal cations, avoiding the additional complexity that would be brought into the system by having several different anions.

Chromium concentration in EPWW is usually reported as a total value, which is the sum of all chromium ions regardless of their oxidation states. The two most stable states of chromium in aqueous solution are Cr(III) and Cr(VI). For the model EPWW, it was decided to only consider Cr(III) by addition of Cr_2_(SO_4_)_3_, in order to be representative of the effluents of modern electroplating sites that have fully replaced Cr(VI) with Cr(III) [36]. This shift has particularly taken place in European production sites after the inclusion of Cr(VI) in the Annex XIV of REACH as a Substance of Very High Concern [37].

#### 2.6.2. Single-Salt Experiments

**Figure 3:**
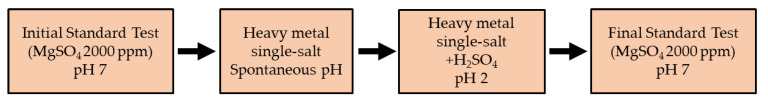
Schema of crossflow filtration experiments performed with the CT at standard conditions with single salts containing heavy metals.

Single-salt filtration tests with each heavy metal were performed at two pH conditions. Twelve coupons were used simultaneously in each experiment. At least 3 coupons of each membrane type were used. The filtration test was performed first without the addition of acid, so that the feed solution would keep the spontaneously occurring pH due to the salt hydrolysis. Then, the pH was decreased to 2 by addition of 10 wt.% H_2_SO_4_. Before and after testing of the heavy metal single salts, the filtration performance of the membrane coupons was characterized at standard conditions at 2000 ppm MgSO_4_. A schema of the series of experiments for each single-salt experiment is shown in Figure 3.

The spontaneously established pH, ionic concentration and ionic strength in each single-salt experiment are shown in Table 5. It should be noted that the goal is not to have the concentrations of heavy metals targeted in the model EPWW, but to be able to compare the single-salt experiments of the different heavy metals among them. For that reason, the ionic strengths of all the single-salt feed solutions are similar, in the order of 10 mmol/L.

#### 2.6.3. Mixed-Salt Experiments

The goal was to achieve the target ionic concentrations presented in Table 3, in order to recreate the desired model EPWW. To do so, the corresponding salts were added to the feed tank, which was finally completed to a final volume of 60 L with DI water. The 12 filtration cells of the CT equipment were filled with 6 coupons of Membrane A and 6 coupons of Membrane B.

The theoretical concentrations, calculated from the known quantities of added salts, are in good agreement with the results of the analysis of the simulated EPWW, as can be seen in Table 6. It should be noted that the term theoretical concentration is used, since the determination of the final volume of the feed (~60 L) is not exact. Therefore, it is reasonable to expect slight differences between the concentrations determined via analytical methods and those values arising from the ratio of mass to volume measurements.

All the cations were determined using inductively coupled plasma optical emission spectroscopy (ICP-OES) and the anions with ion chromatography.

It should be noted that the spontaneously occurring pH of EPWW when all the salts are added to DI water is 3.25. A first filtration test was performed at this condition, while a second test was performed after addition of 10 wt.% H_2_SO_4_ to reach pH 2.

## 3. Analytical Methods

### 3.1. Membrane Characterization

#### 3.1.1. Scanning Electron Microscopy

Scanning electron microscopy (SEM) imaging (Ultra 55 SEM, Carl Zeiss Ltd., Göttingen, Germany) was performed to characterize the morphology of Membrane A. The samples were coated with a thin (30 nm) chromium film using a Z400 sputter system (Leybold, Hanau, Germany).

#### 3.1.2. Membrane Thickness

Membrane thickness was measured using a handheld digital thickness measurement device (Käfer, Germany), with a measuring range of 25 mm, measuring force of 0.65–1.15 N, resolution of 0.001 mm and pretravel of 0.199 mm. The reported mean values with the respective standard deviations results were obtained by measuring the thickness of each membrane at 12 different points.

#### 3.1.3. Electrokinetic Characterization

Streaming potential measurements were performed on each membrane using a SurPASS system (Anton Paar, Graz, Austria) with an adjustable gap cell and KCl 1 mmol/L as electrolyte. pH within the interval 3–9 was adjusted by addition of HCl and NaOH. The zeta potential was calculated through the Smoluchowski equation.

#### 3.1.4. Water Contact Angle

The surface wettability of the membranes was determined using the static water contact angle measurement system PCA 100M/2 (DataPhysics Instruments GmbH, Filderstadt, Germany), by means of the sessile drop method. The image processing software of the equipment automatically recognizes and records the drop contour, as well as the base line at the solid–liquid interface.

For each drop, the mean contact angle results from the average of the left- and right-side angles. At least 5 drops of 5 µL each were deposited in different areas of the membrane coupon. For a given membrane type, 3 different coupons of the same were considered and the reported value corresponds to the average between those 3 coupons. The membranes were tested in dry condition as provided. On the other hand, Membrane C is a TFC RO membrane that is stored wet. Before measuring its water contact angle, Membrane C was left for drying for 24 h at ambient temperature.

### 3.2. Concentration of Ionic Solutes

Different analytical methods were used to determine the concentrations of each ionic species present in the feed and permeate of the filtration experiments, as shown in Table 7. The filtration tests are divided into single-salt and simulated EPWW experiments. In the latter, different analytical methods were used (ICP-OES and ion chromatography) due to the complex nature of the ionic mixture, which could lead to significant interferences if the same methods applied to the single salts were to be used. A more specific discussion of each analytical method is presented in Section 3.2.1, Section 3.2.2, Section 3.2.3, Section 3.2.4 and Section 3.2.5.

#### 3.2.1. Conductivity and pH Measurements

Conductivity and pH were measured using a multi-meter (Schott Prolab 4000, Schott AG, Mainz, Germany) with automatic temperature compensation. Both the conductivity and pH meter probes were regularly calibrated in the full range of measurements to be performed. The concentration of hydronium ions was obtained from the pH measurements. The difference between the hydronium ion concentration and activity was neglected as the activity coefficients in the pH interval under study (pH > 2) are very close to 1 [38].

Calibration curves relating the conductivity and concentration of the following single-salt solutions were obtained at neutral pH: NaCl, ${\mathrm{Na}}_{2}{\mathrm{SO}}_{4}$, ${\mathrm{MgSO}}_{4}$, ${\mathrm{MgCl}}_{2}$,${\;\mathrm{ZnSO}}_{4}$, ${\mathrm{NiSO}}_{4}$,${\;\mathrm{CuSO}}_{4}$. The standard procedure to derive these curves was to determine the salt concentration with an additional analytical method for a series of samples with different concentrations distributed in the interval of interest. Then, the best fitting curve was obtained. In this way, by simply measuring the conductivity of the feed and permeates, the salt concentration in each of them can be determined, so that the observed rejection is calculated.

It should be noted that this approach is valid as long as the feed and permeates keep a pH close to neutrality. For pH < 4, the contribution of hydronium ions to the overall conductivity is no longer negligible, particularly for diluted ionic solutions (characteristic of permeates). Therefore, in all acidic conditions, even for single-salt solutions, another analytical method had to be used to calculate the observed rejection.

#### 3.2.2. Automated Photometric Analyzer

The device Gallery Plus Automated Photometric Analyzer (ThermoFisher Scientific, Dreieich, Germany) was used to determine the concentration of chlorides present in the feed and permeates.

#### 3.2.3. UV–Vis Spectrophotometer

A manual UV–Vis spectrophotometer (Hach Lange DR3900, Düsseldorf, Germany) capable of measuring wavelengths between 320 and 1100 nm was used in this study with the corresponding ready-to-use reagent packages and cuvettes (LCK Kits, Hach Lange Düsseldorf, Germany), in order to determine the concentration of most ions in the single-salt experiments (see Table 7). When necessary, samples were diluted and/or neutralized to meet the required concentration and pH range before measurement.

To avoid interferences of other ionic species in the spectrophotometric determination, Hach Lange cuvettes were only used in single-salt experiments, whereas other methods were chosen for the determination of the complex ionic mixture in the EPWW.

#### 3.2.4. Ion Chromatography

Ion chromatography was performed to measure the concentration of anions in the single-salt experiment with Cr_2_(SO_4_)_3_ and in the simulated EPWW, according to standard ISO 10304-1:2007 (E). Ion chromatograph ICS 1000 (Dionex GmbH) with autosampler AS40 (Thermo Scientific) and a conductivity detector were used. The anions bind to the ion exchange resins in the chromatography column and are eluted based on their charge and size.

#### 3.2.5. Inductively Coupled Plasma Optical Emission Spectroscopy (ICP-OES)

The cation concentrations in the feed and permeate samples of single-salt $Cr_{2}{\left(SO_{4}\right)}_{3}$ and model EPWW were determined by means of ICP-OES (Varian Inc., Vista Pro, CA, USA), following standard ISO 11885:2007 (E). In this method, the samples are injected into argon plasma and excited at high temperature. The atomic emission from the plasma is collected with a lens and imaged in the silt of a wavelength selection device, from which the concentration of the cations is determined.

### 3.3. Determination of MWCO by Gel Permeation Chromatography (GPC)

A gel permeation chromatography system (Tosoh EcoSEC, HLC−8320GPC) equipped with a pre-column (PSS Suprema Pre-column 10 μm, 8 × 50 mm), three columns (PSS Suprema 10 μm, 1000 Å/100 Å/30 Å, 8 × 300 mm) and a refractive index detector was used to obtain the molecular weight distribution of a mixture of PEG molecules present in the feed and permeate samples. An aqueous solution with NaCl 0.1 M was used as mobile phase, containing 0.02 wt.% of sodium azide (NaN_3_) to prevent biofouling inside the GPC system. The eluent flux and temperature were set to 1 mL/min and 45 °C, respectively. The GPC system was calibrated using GPC standards of PEG ranging from 100 to 6000 g/mol. The instrument’s software was used to process and compare the chromatograms, in order to obtain the sieving curves necessary for MWCO determination.

## 4. Results and Discussion

### 4.1. Membrane Morphology

**Figure 4:**
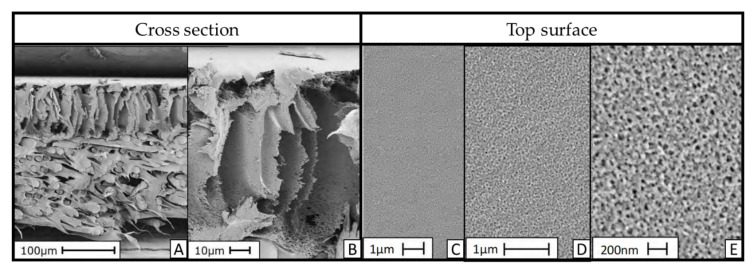
SEM micrographs of Membrane A. (**A**,**B**) Cross-section with different magnifications; (**C**–**E**) top view of the membrane surface.

The micrographs of Membrane A obtained by SEM are presented in Figure 4. The two micrographs on the left (A and B) show the cross-section of Membrane A with two different magnifications. They reveal an asymmetric structure composed of a dense thin top layer on its surface turning into finger-like macrovoids in the membrane bulk. It is also visible that there is no penetration of the membrane into the supporting non-woven fabric. The bulk of the membrane, without considering the non-woven support, has a thickness of ~100 μm, with a dense top layer thickness in the order of a few μm.

The micrographs on the right (C, D and E) show a homogeneous membrane surface. The micrograph with the highest magnification (E) reveals surface nano-porosity with uniformly distributed nano-pores.

### 4.2. Hydrophilicity and Thickness

Water contact angle measurements were performed to determine the degree of hydrophilicity of the membranes used. These results are presented together with those for the measured membrane thicknesses in Table 8.

The thickness of the membrane, as shown in Table 8, includes the supporting non-woven fabrics. Both Membranes A and B use a slightly thicker polypropylene-based non-woven fabric, whereas Membrane C uses a thinner polyester-based non-woven fabric.

The obtained average contact angle experimental data are within the commonly reported relative standard deviation range (of up to 10%) in measurements performed with RO and NF membranes [39]. Since the differences in mean values are statistically significant (according to Student’s *t*-tests with a 95% confidence level performed for Membrane A and B and for Membrane B and C, respectively), the membranes can be ordered in terms of increasing hydrophobicity as follows: Membrane C < Membrane B < Membrane A.

The average water contact angles of Membranes A and B were compared with values reported in the literature and were found to be in good agreement. Mänttäri et al. obtained values between 50° and 60° for the water contact angles of sulfonated NF PES-based membranes [40]. They suggested that for a membrane with a higher contact angle, the sulphonic acid groups are mostly missing from the outside membrane surface and are dominantly present in the membrane bulk material.

As a comparison, Baek et al. systematically studied the water contact angles of different commercial polyamide RO membranes and obtained that most of them are within the range of 20° to 40° [41]. The value determined for Membrane C is within that range, thus confirming its much more hydrophilic surface. It is generally accepted that more hydrophilic membranes favor higher water permeability [42]. For this reason, it would be expected that Membrane B would have a higher water permeability than A. This trend is consistent with the filtration experiments, which showed that the pure water permeability constant of Membranes A and B are 1.26 ± 0.27 and 4.8 ± 1.0 $\frac{L}{m^{2}\cdot h\cdot \mathrm{bar}}$, respectively.

### 4.3. Surface Charge

Another relevant parameter of an NF membrane is its surface charge. NF performance is highly dependent on the interactions established between the membrane surface and the feed solution. The membrane surface chemistry determines which are the ionizable functional groups, whereas the feed pH dictates whether those groups are neutral or not. Besides, possible adsorption of feed ions onto the surface can either neutralize or provide an additional charge to the membrane [43].

**Figure 5:**
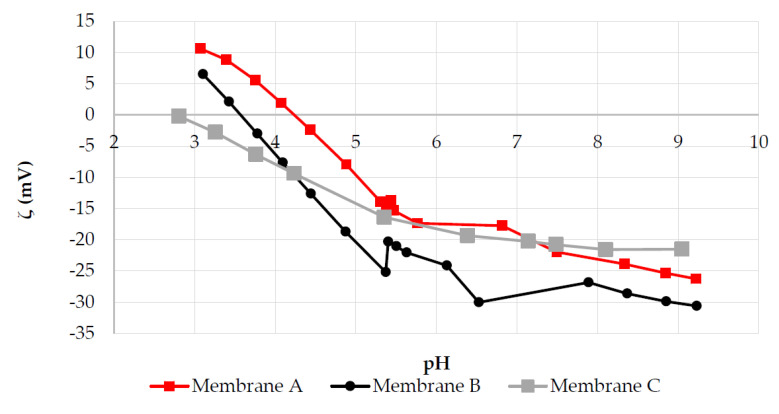
Zeta potential of Membranes A, B and C as a function of pH.

The standard approach to investigate the membrane surface charge is through measuring its zeta potential as a function of pH. The results for the two NF membranes and for the RO membrane under study are shown in Figure 5. It should be noted that the zeta potential determination is restricted to the interval between pH 3 and 9. More extreme pH values would significantly increase the ionic strength of the solution, thus exceeding the acceptable threshold for electrode polarization [44]. In any case, it is reasonable to expect a more positive membrane surface charge as the pH decreases.

The shapes of these zeta potential curves are indicative of amphoteric surfaces. The pH value, at which the zeta potential is null, is referred to as an isoelectric point (IEP). At this pH, it is expected that Donnan exclusion-related effects will be minimal due the zero net surface charge. As can be seen in Figure 5, Membrane A has less negative surface charges than Membrane B at neutral conditions, but more positive surface charges than Membrane B at a pH below ~3.7. The IEP of Membrane A is also slightly higher than that for most commercial NF membranes, which is usually between 3 and 4 [9].

The negative surface charge at higher pH of both Membranes A and B can be explained in terms of the sulfonic acid groups. These membranes are made of hydrophilized PS and sulfonated PES, respectively. The strongly acidic sulfonic acid groups are expected to remain negative over nearly the entire pH range [9]. Therefore, the positive surface charge at low pH is due to protonation of the functional groups of the hydrophilizing agents added to the membrane formulation, which remain under know-how protection.

Another consideration to interpret zeta potential values is that the ionic environment can substantially affect the membrane surface charge. In other words, the zeta potential determined with the electrokinetic analyzer at a given pH with the testing solution (KCl 0.1 M) may not be equal, even at the same pH, in a solution with a different ionic composition [45]. For example, it has been shown that at the same ionic strength and pH 7, the membrane zeta potential is lower in solutions with divalent cations than in solutions with monovalent cations (e.g., MgSO_4_ versus Na_2_SO_4_), which is probably due to preferential adsorption of the divalent species on the negative membrane surface [43]. The nature of the amphoteric behavior of polyamide RO membranes is widely reported in the literature. Since the TFC membranes are made by interfacial polymerization of a monomeric polyamine with a polyfunctional acyl halide, ionizable carboxyl and amine functional groups are present on the membrane surface [46]. Therefore, a positive surface charge results from the protonation of the amine groups, with a pKa varying from 3.6 to 4.6 [47]. On the other hand, the negative charge arises from the deprotonation of the carboxyl groups, with pKa varying from 1.8 to 2.4 [43].

### 4.4. MWCO Dependence on pH

MWCO determination with a mixture of PEG molecules was performed to investigate the contribution of steric hindrance to the overall rejection performance of the NF membranes under study. Besides, the protocol described in Section 2.5 which enables the determination of MWCO values at pH 2 is also useful to reveal potential changes in the membrane structure during filtration at acidic pH.

The series of filtration experiments has already been presented in Figure 2, and the results in terms of salt rejection, MWCO values and permeance are shown in Table 9. The standard deviation values of permeances and MgSO_4_ rejections are calculated considering results obtained with six coupons of each membrane used simultaneously in the crossflow filtration device.

Table 9 shows that the permeance of Membrane A increases in the filtration series, starting at ~1.1 $\frac{L}{m^{2}\cdot h\cdot \mathrm{bar}}$ in the initial standard test and going up to ~1.5 $\frac{L}{m^{2}\cdot h\cdot \mathrm{bar}}$ in the final standard run. The same behavior has been observed in the single-salt experiments with heavy metals. In those experiments, no PEG molecules were used, therefore there is no influence of PEG on the permeance increase in Membrane A. As expected, the membrane with the lower MWCO value (Membrane A) is also the one with the lower permeance.

On the other hand, the permeance of Membrane B decreases in the filtration experiments with PEG. Eventual adsorption of PEG molecules on the membrane surface might have two different effects with contradicting trends: (i) adsorbed PEG molecules could increase the surface hydrophilicity with a positive impact on membrane permeance [48]; and (ii) adsorbed PEG molecules may foul the membrane surface and decrease the water permeation at a given pressure [49]. The latter effect would be dominant for Membrane B according to these experimental results.

**Figure 6:**
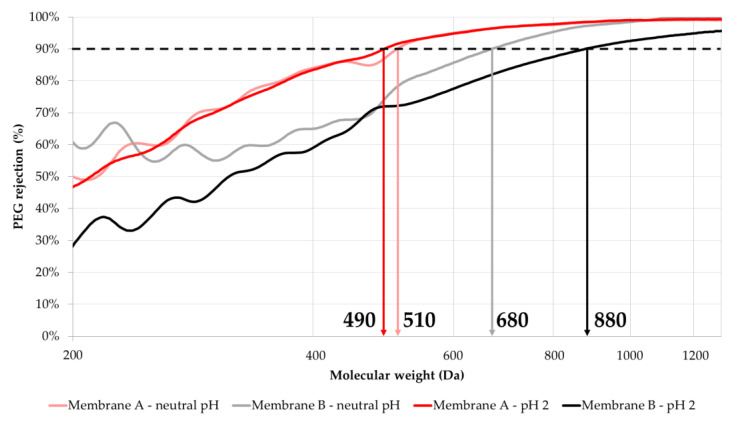
Sieving curves of Membranes A and B at neutral and low pH when filtering a mixture of PEG molecules.

The sieving curves for the determination of MWCO values are presented in Figure 6. It should be noted that the sieving curves are continuous since each of the PEG markers used in the mixture (200/300/400/600/1000/2000/3000 Da) presents a molecular weight distribution, and the markers were chosen so that these distributions would overlap with one another. In this way, there are PEG probes of all molecular weights present in the molecular weight interval of interest.

The effect of pH on the obtained MWCO values can be evaluated comparing the sieving curves. For Membrane A, the operation at acidic pH results in a slight decrease in the MWCO value, from ca. 510 to ca. 490 Da. Despite this reduction, the membrane permeance increases when compared to the filtration of the PEG markers mixture at neutral pH.

On the other hand, when exposed to pH 2, Membrane B shows a very significant increase in its MWCO, which shifts from ca. 680 to ca. 880 Da. Interestingly, this increase in the MWCO is accompanied by a slight reduction in the membrane permeance.

The filtration experiment performed under a pH of 2 has a high ionic concentration arising from the added acid, whereas in the experiment at neutral pH, there are almost no ions present (the conductivity was lower than 10 μS/cm).

The performance of Membrane B can be evaluated by comparing the gray and black sieving curves. The black line represents the PEGs rejection at pH 2 and the gray line at neutral pH. As can be observed, the black line is below the gray line throughout all the MW values, which means that the rejection of the PEG markers of all sizes is decreased by the pH change. However, the distance between the gray and black lines is particularly large at low MW values and this distance decreases as the MW increases.

Luo and Wan [50] summarized and discussed eight possible mechanisms that may be responsible for the variations in membrane flux/permeance and solute rejection induced by pH and salt conditions. Among them, the most frequently assumed are changes in the membrane thickness/porosity ratio and electrostatic repulsion/attraction effects. The latter effects are unlikely to affect (at least directly) the PEGs rejection, as they are electrically neutral molecules. The low pH might have caused a change in Membrane B’s thickness/porosity ratio (e.g., by increasing the porosity and/or average pore size), thus reducing the rejection of the low-molecular weight PEG markers. However, this is a hypothesis which requires further research for its validation, which might not be easily achievable, since, as Luo and Wan [50] have pointed out, one or two mechanisms are generally not enough to explain variations in flux and solute rejection caused by pH. In any case, although, at present, the underlying reasons behind the pH stability of Membrane A and the change in the rejection behavior of Membrane B at low pH are not elucidated, the obtained results clearly demonstrate the importance of characterizing the MWCO of a given membrane at the actual pH conditions of the targeted application.

### 4.5. Filtration of EPWW

#### 4.5.1. Single Salts

Before dealing with the complex EPWW mixture, a series of single-salt experiments were performed with the same heavy metal salts used in the model EPWW. In order to compare the results among the different single-salt experiments, the ionic strength was kept in the same order of magnitude (~10 mmol/L) at a pH condition close to neutrality, and all the salts contained sulfate as the counter-ion of the heavy metals. The precise values have already been presented in Table 5.

The single-salt experiments were performed at two pH conditions: close to neutrality and pH 2. The former condition close to neutrality is not exactly pH 7, but rather the spontaneous pH arising from the dissolution of the salt in DI water. Multivalent cations such as Ni(II), Cu(II), Zn(II) and Cr(III) are very polarizing due to the great concentration of positive charge in a small volume. In aqueous solution, they behave as Lewis acids, strongly interacting with the O-H bond in water and eventually forming a complex with hydroxide anions. Protons are released because of this complexation, triggering a decrease in the water pH. As can be seen in Table 5, the pH drop is particularly significant for Cr(III) (pH 3.23), which is consistent with the higher concentration of charge in the trivalent cation. For the other cations, the pH remains above 5. It was deliberately decided not to adjust the pH to 7, since doing so would imply adding NaOH. The Na^+^ ions could interfere with the study of the rejection of the heavy metal cations.

**Figure 7:**
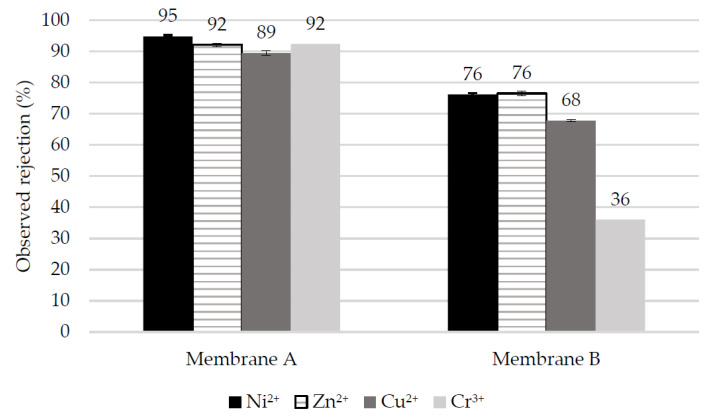
Observed rejection of multivalent cations in single-salt tests conducted at spontaneous pH.

Figure 7 shows the rejection performance of the NF membranes during the filtration of the heavy metal single-salt solutions. It is evident that Membrane A exhibits higher metal rejection for all of the tested compounds compared to Membrane B.

While Membrane A presents a high rejection of more than ca. 90% for all cations, the rejection for Membrane B drops to ca. 68% for Cu(II), and even to only 36% for Cr(III). For all divalent cations, the feed has a pH above 5, which means that both membranes have a negative zeta potential and that sulfate is the dominant ion in terms of rejection. However, the feed solution with Cr(III) is at pH 3.23, which is very close to the IEP of Membrane B. Therefore, a very poor contribution of Donnan exclusion is to be expected for Membrane B, thus relying on Cr(III) rejection mainly by steric and dielectric exclusion. It has already been shown that Membrane B is a quite loose NF membrane with an MWCO that increases at pH extremes. On the contrary, Membrane A, with an IEP above pH 4, shows a much higher Cr(III) rejection. At pH 3.23, the zeta potential of Membrane A is quite high and positive (+8 mV, as shown in Figure 5), promoting exclusion of highly charged cations such as Cr(III).

**Figure 8:**
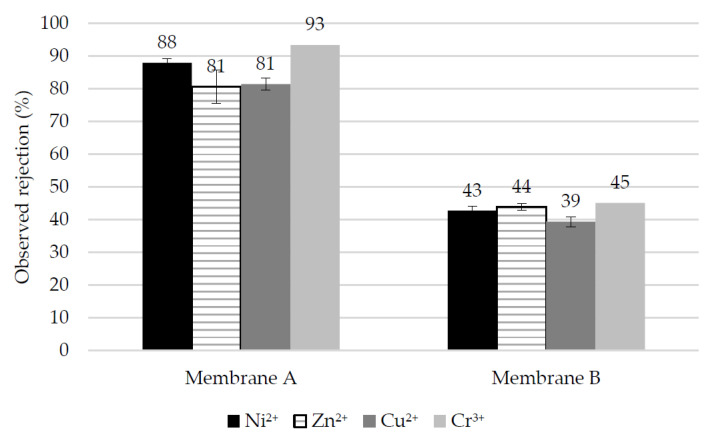
Observed rejection of multivalent cations in single-salt tests conducted at pH 2.

When the pH is decreased to 2 by the addition of sulfuric acid, the performance presented in Figure 8 is observed. Membrane A slightly decreases their retention except for the case of Cr(III). It should be noted that at pH 2, ionic rejection of divalent salts is no longer controlled by sulfates as in Figure 7, but by the multivalent cations, due to the inversion in the sign of the membrane surface charge. It is reasonable that trivalent Cr(III) is more strongly rejected than the divalent cations, as both Donnan and dielectric exclusion increase with the ion valence [51].

**Figure 9:**
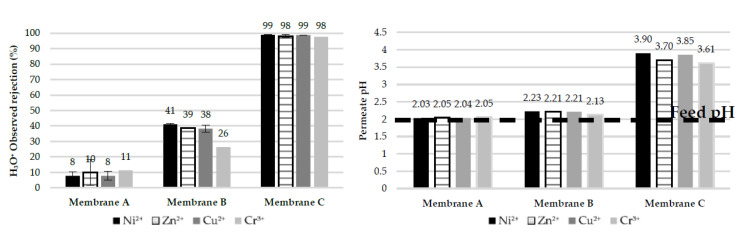
(**Left**) Observed rejection of hydronium ions in single-salt tests at pH 2. (**Right**) Permeate pH.

Figure 9 displays the hydronium ion observed rejection together with the shift in pH between the feed and permeate. As regards the NF membranes, divalent cations rejection for Membrane A is linked to the high permeation of protons, whereas the opposite holds for Membrane B. In the case of the RO membrane (Membrane C), it shows a high proton rejection, reflected by the relatively high pH of the permeates (pH > 3.6) compared to the feed (pH = 2). Unlike Membrane A, Membrane C simultaneously shows a high rejection of metal cations and protons. This fact reveals a very different behavior of RO in the treatment of EPWW, which does not enable the recovery of concentrated acid in the permeate side.

**Figure 10:**
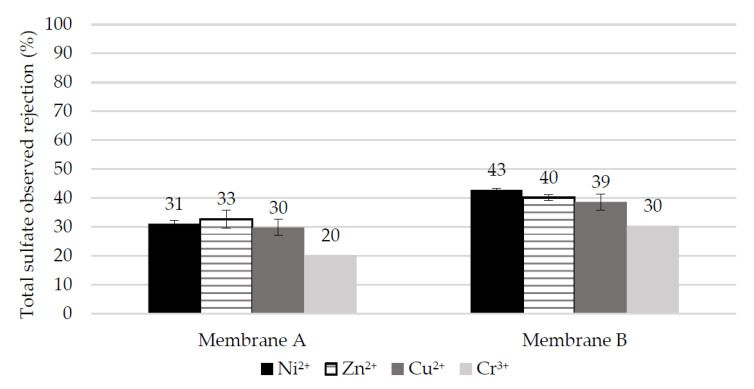
Observed rejection of total sulfate in single-salt experiments at pH 2.

One possible explanation for the high proton rejection of Membrane C is related to the anion rejection. Membrane C rejects both metal cations and sulfates to a large extent (~100%), meaning that the hydronium ions do not have any counter-ion to permeate with. Therefore, even though they are small enough to permeate through the RO membrane, their permeation is restricted by the electroneutrality requirement. On the other hand, the asymmetry in metal cations (Figure 8) and anion (sulfate) (Figure 10) rejection in the case of NF membranes explains their hydronium ions permeation.

#### 4.5.2. Electroplating Mixture

Filtration experiments for the two NF membranes were also performed with the model EPWW solution, at both spontaneous pH (pH = 3.25) and at pH 2 after the addition of sulfuric acid. The observed rejection results are presented in Table 10.

As expected, the monovalent ions (Na^+^ and Cl^−^) easily permeate through both NF membranes, whereas divalent cations and anions are more strongly rejected. The retention of heavy metal cations is slightly lower than in the single-salt experiments, for Membranes A and B at the two pH conditions. This is likely due to the increased ionic strength of the EPWW mixture (59 meq/L at pH 3.3 and 73.1 meq/L at pH 2) when compared to the feeds in the single-salt experiments (~10 meq/L at spontaneous pH and 21 meq/L at pH 2). The increased ionic strength shrinks the electrical double layer, resulting in lower charge exclusion. Besides, permeation of monovalent anions like chloride can also hinder the divalent cation rejection.

The decrease in pH of the EPWW triggers a significant increase (>10%) in the rejection of all heavy metal cations by Membrane A, whereas Membrane B only improves its rejection of Ni(II) and Cr(III). On the other hand, the rejection of chloride by both NF membranes barely changes with pH, while the retention of sulfates decreases at pH 2, as a consequence of sulfate speciation into bisulfate and the higher positive zeta potential of the membranes. Several authors have also reported that speciation into bisulfate is responsible for the lower sulfate rejection as the pH is decreased [30,52]. Lopez et al. argued that the transport of single-charged ions (such as ${\mathrm{HSO}}_{4}^{-}$) is favored over that of multi-charged ions by means of the dielectric exclusion phenomena [21].

Finally, the quality of the permeates of the EPWW at pH 2 is compared with the legally binding maximum concentration levels of wastewater prescribed by the United States Environmental Protection Agency and shown in Table 11. Unfortunately, none of the NF permeates can meet the discharge requirements, being still one order of magnitude higher. Therefore, hybrid processes to achieve further purification of the NF permeates should be considered, such as coupling NF with a downstream cation-exchange resin [53]. The operational time of the ion-exchange resin between regeneration cycles would be significantly extended, due to the lower load of heavy metals in the NF permeate.

## 5. Conclusions

The present work has characterized a novel pH-stable NF membrane (Membrane A) and compared its characteristics and filtration performance with a commercial pH-stable NF membrane (Membrane B), using both heavy metal (Ni, Zn, Cu and Cr) single-salt solutions as well as a simulated EPWW mixture. A standard RO membrane (Membrane C) was also included in some experiments for comparison. The following are the main outcomes of the study:

- SEM images of Membrane A reveal an asymmetric polymeric structure composed of a dense few μm-thick thin top layer on its surface, turning into finger-like macrovoids in the membrane bulk. The membrane has a thickness of ca. 100 μm without considering the non-woven fabric support. The top surface shows uniformly distributed nano-pores.
- Membrane A is thicker with an average thickness of ca. 261 μm versus ca. 229 μm of Membrane B. Membrane A is more hydrophobic (water contact angle of 64.3°) than Membrane B (water contact angle of 53.6°). These values are comparable to those reported in the literature and are consistent with the lower pure water permeability of Membrane A compared to that of Membrane B (1.26 ± 0.27 versus 4.8 ± 1.0 $\frac{L}{m^{2}\cdot h\cdot \mathrm{bar}}$).
- The IEP of Membrane A (at a pH of ca. 4.2) is higher than that of Membrane B (at a pH of ca. 3.5) and Membrane A has more positive surface charges than Membrane B in the pH range of 3 to 3.5.
- Membrane A has an improved low pH stability, showing only a slight change in its MWCO value, from ca. 510 to ca. 490 Da, compared to a significant MWCO shift for Membrane B from ca. 680 to ca. 880 Da. This means that Membrane A barely changes its rejection performance in terms of neutral molecules such as PEGs, while Membrane B becomes significantly more permeable to low-molecular weight (MW < ca. 490 Da) PEGs fractions, thus demonstrating the utmost importance of MWCO determination at the pH value of the targeted application.
- Membrane A shows much higher (by two to three times) rejection of heavy metals compared to Membrane B at the spontaneous pH of 3.25 of the simulated EPWW mixture which becomes even higher (by > 10%) at a pH of 2, which can be attributed to the higher positive surface charge of Membrane A at low pH. Membrane A also enables a higher hydronium ions concentration in the permeate than Membrane B, which is due to the more pronounced Donnan exclusion effects for the cations at the solution pH below the membrane IEP.

The rejection performance of the hereby studied pH-stable NF membranes shows that they cannot be used as a stand-alone process to treat EPWW if the goal is to obtain a permeate that meets the discharge limits for heavy metals. For such a purpose, hybrid processes or RO should be applied. However, this drawback could be offset by the enhanced acid recovery of which Membrane A is capable. This is an interesting feature, which can be utilized to improve the process economy and sustainability, as the purified acidic permeate could be recycled into the process, thus reducing the consumption of fresh acid and closing the loop of the acidic permeate.

## Figures and Tables

**Table 1:** Main characteristics of commercial pH-stable nanofiltration (NF) membranes (testing conditions can be found in the corresponding references).

Model	Material	Configuration	pH Range	Permeance ($\frac{L}{m^{2}\cdot h\cdot \mathrm{bar}}$)	Rejection (%)				Max T (°C)	Drawback	References
					MWCO	NaCl	MgSO_4_	Na_2_SO_4_			
**Microdyn-Nadir** **NP010**	Sulfonated PES	Asymmetric	0–14 *	>5 *	1000–1200 *	-	-	35–75 *	-	High MWCO	[7]
**Microdyn-Nadir** **NP030**	Sulfonated PES	Asymmetric	0–14	1.7	520	30	-	80–95*	95	Low flux	[3,8,9,10]
**Hydranautics/Nitto Denko** **HYDRA-CoRe 70pHT**	Sulfonated PES	TFC	1–13.5 *	5.8	720 *	70	-	97.6	70 *	High NaCl rejection	[11,12]
**Osmonics/GE/Suez Duracid**	Proprietary	TFC	0–10	7 *−8	400	-	98 *	-	70 *	Only for acid	[10,13]
**Koch SELRO** **MPS−34**	Proprietary	Composite	0–14	1.75	200 *−300	35	-	-	50–70 *	Low flux	[10,14,15]
**Koch SELRO** **MPS−36**	Proprietary	Composite	0–14	8 *	1000	10 *	-	-		High MWCO	[3,16]
**Dupont Filmtec** **NF−270**	Polyamide	TFC	3–10 *	10.6	200–400	-	>97 *	-	45 *	Only mild pH	[3,17]
**Inopor** **Nano**	TiO_2_ (α-Al_2_O_3_ support)	Composite	0–14	8.6	750	-	-	-	350	Expensive and low packing density	[18]
				0.77	450	-	-	-	350		[3,18]

*: Value indicated by manufacturer in product specification.

**Table 2:** Polymeric pH-stable membranes developed in recent years.

Material	pH Range	Permeance ($\frac{L}{m^{2}\cdot h\cdot \mathrm{bar}}$)	MWCO (Da)	Rejection (%)				IEP (pH)	Year	Reference
				NaCl	MgSO_4_	Na_2_SO_4_	MgCl_2_			
TCF with PS as support, SPEEK interlayer and polysulfonamide active layer	0–7(Alkaline stability not reported)	1.74	800	89	97	99.7	79	4.1	2020	[4]
Polyvinylidene fluoride grafted with polystyrene sulfonic acid	0–14	2.4	<500	60	80	-	-	<3	2018	[10]
IP of 1,3,5-(tris-piperazine)-triazine and trimesoyl chloride on top of PS UF support	>1	9	-	40	97	98.6	-	3.5	2018	[19]
Poly(vinyl alcohol)-aminopropyl triethoxysilane	0–14	0.7	-	50–55	-	98.5	-	-	2014	[12]
Polyamine on porous PES support	1–13	2.5	500	65	40	-	90	7.5	2014	[3]
SPEEK on PES UF support	1–13	4.5	500	60	-	90	-	<2	2011	[20]

**Table 3:** Summary of chemicals used in this study.

Chemicals	Manufacturer	Purity
MgSO_4_∙7H_2_O	Fluka Honeywell	≥98%
CaSO_4_∙2H_2_O	Sigma Aldrich	≥99%
NaCl	VWR	100%
Na_2_SO_4_	Sigma Aldrich	≥99%
HCl	Merck	37%
NaOH	Merck	49–51%
H_2_SO_4_	Merck	95–97%
NiSO_4_∙6H_2_O	Alfa Aesar	>98%
Cr_2_(SO_4_)_3_∙H_2_O	Sigma Aldrich	For synthesis
ZnSO_4_∙7H_2_O	Alfa Aesar	>98%
CuSO_4_∙5H_2_O	Merck	For analysis
PEG 200/300/400/600/1000/2000/3000 MW	Merck	For synthesis

**Table 4:** Composition of real electroplating wastewater (EPWW) and target concentration in simulated EPWW.

Ion		Wang 2007 [26]	Wei 2013 [27]	Target of This Work
Cations (ppm)	Na^+^	13.8	653.8	657
	K^+^	1.9	105.7	---
	NH_4_^+^	34.0	---	---
	Ca^2+^	16.0	76.9	77
	Mg^2+^	0.2	19.9	20
	Zn^2+^	0.6	14.8	15
	Mn^2+^	0.1	---	---
	Ni^2+^	0.8	146.7	147
	Cu^2+^	11.8	57.8	58
	Total chromium	17.1	123.5	124
Anions (ppm)	F^−^	232.5	---	---
	Cl^−^	27.5	943.8	1013
	NO_3_^−^	100.8	64.8	---
	NO_2_^−^	---	38.5	---
	HCO_3_^−^	---	---	---
	SO_4_^2−^	415.8	971.0	957
pH		2.32	2.20	2.00

**Table 5:** pH, concentration and ionic strength of each single-salt feed before the addition of acid.

Heavy Metal	Salt	pH	Feed Concentration at Spontaneous pH			Feed Ionic Strength (mmol/L)
			Metal (ppm)	Metal (mmol/L)	Sulfate (mmol/L)	
Cu^2+^	CuSO_4_	5.05	145	2.3	2.3	9.2
Zn^2+^	ZnSO_4_	5.60	153	2.4	2.4	9.6
Ni^2+^	NiSO_4_	5.70	135	2.3	2.3	9.2
Cr^3+^	Cr_2_(SO_4_)_3_	3.23	50	1.0	1.5	7.5

**Table 6:** Comparison of target and measured values of EPWW at the two pH conditions.

Ion	Feed pH 3.25		Feed pH 2	
	Theoretical Amount (ppm)	Result of Analysis (ppm)	Theoretical Amount (ppm)	Result of Analysis (ppm)
Na^+^	657	690	657	680
Ca^2+^	77	78	77	80
Mg^2+^	20	21	20	22
Zn^2+^	15	16	15	16
Ni^2+^	147	150	147	160
Cu^2+^	58	56	58	59
Cr^3+^	124	93	124	95
Cl^−^	1013	900	1013	910
SO_4_^2−^ total	957	820	1801	1650

**Table 7:** Analytical methods used to determine ionic concentrations. LCK is the commercial name of the ready-to-use kits from Hach Lange.

		Single-Salt		Model EPWW
		Spontaneous pH	pH 2	Both pH Conditions
Cations	Na^+^	Conductivity	Charge balance	ICP-OES
	Ca^2+^	Conductivity	UV–Vis (LCK-327)	
	Mg^2+^	Conductivity	UV–Vis (LCK-326)	
	Zn^2+^	Conductivity	UV–Vis (LCK-360)	
	Ni^2+^	Conductivity	UV–Vis (LCK-337)	
	Cu^2+^	Conductivity	UV–Vis (LCK-329)	
	Cr^3+^	ICP-OES	ICP-OES	
Anions	Cl^−^	Conductivity	Automated photometric analyzer	Ion chromatography
	SO_4_^2−^	Conductivity	UV–Vis (LCK−153)	

**Table 8:** Water contact angle and membrane thickness.

Membrane	Contact Angle (°)		Thickness (μm)	
	Mean Value	Standard Deviation	Mean Value *	Standard Deviation
A	64.3	3.9	261	5
B	53.6	5.8	229	6
C	36.4	4.0	144	3

* Including the supporting non-woven fabrics.

**Table 9:** Evolution of membranes’ performance in terms of salt rejection, molecular weight cut-off (MWCO) and permeance throughout experiments.

Membrane	Parameter	Initial Standard pH 7	PEG pH 5.35	PEG pH 2	Final Standard pH 7
A	MgSO_4_ Rejection (%)	93.2 ± 0.6	---	---	89.0 ± 0.4
	MWCO (Da)	---	ca. 510	ca. 490	---
	A ($\frac{L}{m^{2}\cdot h\cdot \mathrm{bar}}$)	1.07 ± 0.04	1.25 ± 0.01	1.40 ± 0.05	1.57 ± 0.05
B	MgSO_4_ Rejection (%)	66.3 ± 0.8	---	---	61.6 ± 0.6
	MWCO (Da)	---	ca. 680	ca. 880	---
	A ($\frac{L}{m^{2}\cdot h\cdot \mathrm{bar}}$)	4.04 ± 0.14	3.64 ± 0.10	3.56 ± 0.09	3.75 ± 0.09

**Table 10:** Composition of EPWW at spontaneous and acid pH, together with observed rejection and permeance values for each membrane and pH and conductivity of each sample.

Ions	Electroplating Mixture					
	Spontaneous pH			pH 2 (+H_2_SO_4_)		
	C Feed	Membrane Rejection (%)		C Feed	Membrane Rejection (%)	
	mmol/L	A	B	mmol/L	A	B
Na^+^	30	29	20.3	29.6	27.9	14.7
Ca^2+^	1.9	66.7	32.1	2	77.5	28.8
Mg^2+^	0.9	66.2	28.6	0.9	79.5	27.3
Zn^2+^	0.2	65	31.3	0.2	76.3	25
Ni^2+^	2.6	68	26.7	2.7	80	31.3
Cu^2+^	0.9	60.7	32.1	0.9	72.9	30.5
Cr^3+^	1.8	81.7	32.3	1.8	87.4	43.2
Cl^−^	25.4	14.4	0	25.7	14.3	2.2
SO4^2−^ total	8.5	90.5	59.8	17.2	58.8	39.4
	**Feed**	**Perm A**	**Perm B**	**Feed**	**Perm A**	**Perm B**
pH	3.3	3.5	3.4	2	2	2.08
*K* (µS/cm)	4500	3000	3700	8500	7100	7200
Permeance ($\frac{L}{m^{2}\cdot h\cdot \mathrm{bar}}$)	---	0.81 ± 0.19	5.24 ± 0.4	---	0.84 ± 0.19	4.73 ± 0.37

**Table 11:** Comparison between heavy metal concentration in permeates and Maximum Contaminant Levels of the EPA.

Heavy Metal	Concentration (ppm)			
	Discharge Limit [33]	Model EPWW	Permeate A	Permeate B
Chromium	0.05	95	12	54
Copper	0.25	59	16	41
Nickel	0.2	160	32	110
Zinc	0.8	16	4	12

## Abbreviations

Abbreviations used are arranged alphabetically.

CA	Cellulose acetate
CT	Coupon tester
DI	Deionized water
EPWW	Electroplating wastewater
GPC	Gel permeation chromatography
ICP-OES	Inductively coupled plasma optical emission spectroscopy
IEP	Isoelectric point
IP	Interfacial polymerization
MWCO	Molecular weight cut-off
NF	Nanofiltration
PEG	Polyethylene glycol
PES	Polyethersulfone
PS	Polysulfone
RO	Reverse osmosis
SEM	Scan electron microscopy
SPEEK	Sulfonated poly(ether ether ketone)
TFC	Thin film composite
UF	Ultrafiltration

## Symbols and Nomenclature

Symbols are shown in order of appearance.

**Symbols**	**Parameter**	**SI Units**
***P***	Pressure	Pa
**Δ*****P***	Pressure gradient	Pa
**$R_{i,\;observed}$**	Observed rejection of component i	−
**$c_{p,i}$**	Molar concentration of component i in permeate	$\mathrm{mol}\cdot m^{-3}$
**$c_{f,i}$**	Molar concentration of component i in feed	$\mathrm{mol}\cdot m^{-3}$
**$J_{V}$**	Volumetric flux	$m^{3}\cdot m^{-2}\cdot s^{-1}$
**V**	Volume	$m^{3}$
**$A_{M}$**	Area of membrane filtration	$m^{2}$
**t**	Time of permeate collection	s
***Π***	Osmotic pressure	Pa
**$C_{i}$**	Molar concentration of component i	$\mathrm{mol}\cdot m^{-3}$
***R***	Ideal gas constant	$J\cdot K^{-1}\cdot {\mathrm{mol}}^{-1}$
***T***	Temperature	K
***K***	Conductivity	$S\cdot m^{-1}$
